# *HLA* Allele E*01:*01* Is Associated with a Reduced Risk of EBV-Related Classical Hodgkin Lymphoma Independently of HLA-A*01/*02

**DOI:** 10.1371/journal.pone.0135512

**Published:** 2015-08-11

**Authors:** Paloma Martín, Isabel Krsnik, Belen Navarro, Mariano Provencio, Juan F. García, Carmen Bellas, Carlos Vilches, Natalia Gomez-Lozano

**Affiliations:** 1 Group of Molecular Pathology, Instituto de Investigación Sanitaria Puerta de Hierro (IDIPHIM), Majadahonda, Spain; 2 Department of Hematology, Instituto de Investigación Sanitaria Puerta de Hierro (IDIPHIM), Majadahonda, Spain; 3 Department of Oncology, Instituto de Investigación Sanitaria Puerta de Hierro (IDIPHIM), Majadahonda, Spain; 4 Department of Pathology, MD Anderson Cancer Center, Madrid, Spain; 5 Group of Immunogenetics and Histocompatibility, Instituto de Investigación Sanitaria Puerta de Hierro (IDIPHIM), Majadahonda, Spain; 6 Group of Immunity and lymphoproliferative diseases, Instituto de Investigación Sanitaria Puerta de Hierro (IDIPHIM), Majadahonda, Spain; The University of North Carolina at Chapel Hill, UNITED STATES

## Abstract

**Background:**

An inefficient immune response against Epstein-Barr virus (EBV) infection is related to the pathogenesis of a subgroup of classical Hodgkin lymphomas (cHL). Some EBV immune-evasion mechanisms target HLA presentation, including the non-classical HLA-E molecule. HLA-E can be recognized by T cells via the TCR, and it also regulates natural killer (NK) cell signaling through the inhibitory CD94/NKG2A receptor. Some evidences indicate that EBV-infected B-cells promote the proliferation of NK subsets bearing CD94/NKG2A, suggesting a relevant function of these cells in EBV control. Variations in CD94/NKG2A-HLA-E interactions could affect NK cell-mediated immunity and, consequently, play a role in EBV-driven transformation and lymphomagenesis. The two most common *HLA-E* alleles, E*01:*01* and E*01:*03*, differ by a single amino acid change that modifies the molecule function. We hypothesized that the functional differences in these variants might participate in the pathogenicity of EBV.

**Aim:**

We studied two series of cHL patients, both with EBV-positive and-negative cases, and a cohort of unrelated controls, to assess the impact of *HLA-E* variants on EBV-related cHL susceptibility.

**Results:**

We found that the genotypes with at least one copy of E*01:*01* (i.e., E*01:*01* homozygous and heterozygous) were underrepresented among cHL patients from both series compared to controls (72.6% and 71.6% vs 83%, p = 0.001). After stratification by EBV status, we found low rates of E*01:*01*-carriers mainly among EBV-positive cases (67.6%). These reduced frequencies are seen independently of other factors such as age, gender, HLA-A*01 and HLA-A*02, *HLA* alleles positively and negatively associated with the disease (adjusted OR = 0.4, p = 0.001). Furthermore, alleles from both *HLA* loci exert a cumulative effect on EBV-associated cHL susceptibility.

**Conclusions:**

These results indicate that E*01:*01* is a novel protective genetic factor in EBV-associated cHL and support a role for HLA-E recognition on the control of EBV infection and lymphomagenesis.

## Introduction

Epstein-Barr virus (EBV) is a ɣ-herpesvirus with a high prevalence worldwide (over 90%) which interacts with the host immune system and typically establishes benign lifelong latent infection in the B-cells of healthy immunocompetent individuals [1]. However, in some cases, the normal homeostasis of the EBV-specific response is disrupted and leads to the malignant transformation of the infected cells. EBV-driven oncogenesis is related to the development of a subset of several B-cell lymphomas in immunocompetent individuals (mainly, Burkitt [BL], diffuse large B cell [DLBCL] and classical Hodgkin’s [cHL] lymphomas) and in immunocompromised patients (post-transplant [PTLD] and HIV-associated lymphoproliferative disorders).

The relationship between EBV infection and cHL is well established. The rates of EBV-related cHL vary by ethnic group, age, gender, socio-economic status, and histological subtype, and its development is associated with a previous history of infectious mononucleosis [2]. The epidemiology of EBV suggests a complex interplay between genetic, viral and environmental factors that converge to an inadequate immune control of EBV infection. However, genetic drivers of EBV-specific immune responses and lymphomagenesis in EBV-related cHL remain unclear.

There is increasing evidence that the NK cell repertoire plays a role in EBV-directed immunity. In this way, NK cells exhibit greater cytotoxicity during acute EBV infection, and in a humanized mouse model, NK cells limit the EBV viral load, infectious mononucleosis symptoms, and tumor formation [3–5]. Their significance in the control of EBV infection is supported by the observation that primary immunodeficiencies affecting NK cells or NK cell recognition of EBV-transformed cells are associated with EBV-positive malignancies and fatal EBV infection [6–8].

Recently, an EBV-driven imprint on the NK compartment has been reported and is characterized by the proliferation of an NK cell population expressing the inhibitory receptor CD94/NKG2A (CD56^dim^NKG2A^+^KIR^-^CD57^-^) in tissues and peripheral blood during acute symptomatic infection [9, 10]. Moreover, a CD56bright NK cell subset with a similar NKG2A+ phenotype proved to be relevant for controlling infection in the tonsils and to have the potential to inhibit in vitro EBV-induced transformation [11]. These findings support a role for CD94/NKG2A-HLA-E signaling in NK-mediated control of EBV and its associated pathologies.

The inhibitory CD94/NKG2A receptor, a member of the heterodimeric CD94/NKG2 family, is expressed on NK and CD8+ T cell subsets. This receptor interacts with a high affinity and in a peptide-specific manner with the non-classical class I molecule HLA-E loaded with signal peptides derived from other HLA-I molecules [12, 13]. The CD94/NKG2A-HLA-E interaction constitutes a sensor that monitors HLA class I expression on healthy cell surfaces and provides an immunomodulatory mechanism of tolerance. HLA‐E can also present stress- or pathogen-derived peptides (including EBV), whether to CD94/NKG2A or TCR bearing cells, to survey cellular stress or infection by NK and T cells [14–16].

Expression of HLA molecules on infected cells varies during the EBV cycle infection and inversely correlates with sensitiveness to NK killing. Latently EBV-infected B-lymphoblastoid cell lines (B-LCLs) express high levels of HLA molecules, and resist NK lysis [3, 17]; however, HLA class I expression is down-modulated upon entry into the lytic cycle, which is associated with an increase of the NK cell response [18, 19]. HLA class I antigens synthesis and assembly pathways are disrupted in a concerted manner by several EBV antigens (e.g., BNLF2a, BGLF5, BILF1, vIL10) during the lytic cycle, which affects and targets a broad range of HLA antigens [20–22]. HLA-E expression is also affected by EBV immune-evasion mechanisms, presumably circumventing the recognition of HLA-E-viral peptide complexes by CD8+ T cells [23].

Compared to the characteristic genetic diversity of classical class I *MHC* genes, *HLA-E* is fairly conserved. The low level of *HLA-E* polymorphism was present before the generation of classical *HLA* diversity and seems to be driven by a different selective pressure. These features suggest an essential function for this molecule [24, 25]. Fifteen different *HLA-E* nucleotide sequences have been described to encode two different protein sequences: E*01:01 and E*01:03 (http://www.ebi.ac.uk/ipd/imgt/hla [26]). These two HLA‐E molecules are present in more than 90% of the population with similar frequencies, suggesting that balancing selection is acting on the dimorphism [27]. Both variants differ at only one non-synonymous substitution (1147A>G; rs1264457) that results in an amino acid change (arginine to glycine) at position 107 in the alpha-2 domain of the heavy chain. This substitution confers different levels of surface expression and peptide affinities. E*01:03 is expressed at higher levels than E*01:01, probably due to an increase in peptide binding ability that results in a more stable configuration [28].

The allelic diversity of *HLA-E* is associated to several clinical situations. In HCV infection, the homozygous E*01:*03* genotype seems to correlate with chronification [29], and in hematopoietic stem cell transplantation, it has been shown to confer protection against graft‐versus‐host disease, leukemia relapse and mortality [30–32]. Other investigators have reported that the E *01:*01* allele associates with recurrent miscarriage [33] and a reduced risk of Behcet’s disease [34].

We performed a case-control study to evaluate whether the genetic diversity of *HLA-E* affects susceptibility to lymphomagenesis in cHL and its relationship to EBV infection.

## Materials & Methods

### Ethics Statement

The project was approved by the Ethics Committee of Hospital Puerta de Hierro (Approval number: Acta n° 304). Written informed consent was provided by all of the patients and controls.

### Patients and clinicopathological data

One hundred and seventy-five patients diagnosed of cHL (73 EBV+ and 102 EBV-) and 400 unmatched healthy individuals from Hospital Puerta de Hierro Majadahonda (Madrid, Spain) were enrolled in this study. Another series of 82 patients diagnosed with cHL (35 EBV+ and 47 EBV-) from MD Anderson Cancer Center (Madrid, Spain) were studied to validate the results.

cHL was diagnosed based on a morphological and immunohistochemical examination of biopsy materials using standard techniques. The histopathological classification and cHL subclassification were based on the World Health Organization (WHO) 2008 criteria as follows: nodular sclerosis (NS), mixed cellularity (MC), lymphocyte-rich (LR), lymphocyte-depleted (LD) or not classified (NC) cHL. HIV-positive patients were excluded from the study.

Nuclear staining of HRS by in-situ hybridization for EBERs using the Bond ISH EBV-EBER probe was used to detect EBV-infection in the cells. The main characteristics of the cHL patient are summarized in Table 1, and the other clinico-pathological parameters of the patients are shown in S1 Table.

**Table 1:** Characteristics of the cHL patients and control subjects.

% (N)	All cHL (N = 257)		EBV+ cHL (N = 108)		EBV- cHL (N = 149)		Controls (N = 400)
	*HPH (N = 175)*	*MDA (N = 82)*	*HPH (N = 73)*	*MDA (N = 35)*	*HPH (N = 102)*	*MDA (N = 47)*	
*Male gender*	56.8 (107)	56.8 (46)	79.5 (58)	57.1 (20)	48.0 (49)	55.3 (26)	47.5 (190)
*Age* <45 years	69.7 (122)	64.2 (52)	52.1 (38)	51.4 (18)	82.4 (84)	72.3 (34)	60.3 (240)
*Histological subtype*							
*NS*	60.6 (106)	61.0 (50)	43.8 (32)	45.7 (16)	72.5 (74)	72.3 (34)	
*MC*	30.3 (53)	29.3 (24)	45.2 (33)	37.1 (13)	19.6 (20)	23.4 (11)	
*Other*	4.6 (8)	3.7 (3)	5.5 (4)	8.6 (3)	3.9 (4)	0	
*NC*	4.6 (8)	6.1 (5)	5.5 (4)	8.6 (3)	3.9 (4)	4.3 (2)	

HPH, patients from the Hospital Puerta de Hierro. MDA, patients from MD Anderson Cancer Center. NC: not classified.

### *HLA* typing

DNA was extracted from either peripheral blood with DNAzol (MRC, Cincinnati, OH) or using a Maxwell 16 Blood DNA purification kit (Promega Corp. Madison, CA); or from the paraffin-embedded tissue of biopsied tumoral lymph nodes using the QIAmp DNA FFPE Tissue Kit (Qiagen, Hilden, Germany) according to the manufacturer’s instructions.

*HLA-E* typing discriminating the single nucleotide polymorphism in codon 107 (A>G) was performed using PCR-SSP as described [35] with minor modifications. To validate the genotyping method, reference DNAs from the IHW cell panel were used. Randomly selected samples and problematic DNAs were examined by DNA sequencing in a 3130xl Genetic Analyzer Sequencer (Applied Biosystems, Foster City, CA, USA) using the amplification primers HLA-E_Fcon290 5’-ACCGCACAGATTTTCCGAGT-3’ and HLA-E_Rcon396 5’-AGGCGAACTGTTCATACCCG, with HLA-E_Fi6 5’-TCAGTTTAGGCCAAAATGCCCA-3’ as an internal primer for sequencing. HLA-A*01 and A*02 typing was performed by PCR-SSP with the primer pairs FHc527 5’-TGGGAGGCGGTCCATGC-3’ and Rhcg570 5’-AGGTATCTGCGGAGCCCG-3’ for A*01, and FHa144 5’-GAGCCCCGCTTCATCGCA-3’ and RHagg256 5´-TTCACTTTCCGTGTCTCCCC-3’ for A*02 (details available upon request).

### Statistical Analysis

Differences in the distribution of *HLA-E* genotypes reported in this study were calculated using the Pearson χ^2^ test and considering a dominant model for the protective allele HLA-E*01:*01* (E*01:*01* carriers vs negatives). In both the control and patient groups, *HLA-E* genotypes were evaluated for adequacy to Hardy-Weinberg equilibrium.

Odds ratio (OR) values and 95% confidence intervals (CIs) for relative risks were calculated for the genotypes. Frequencies of combined *HLA-A-HLA-E* phenotypes were compared with Pearson χ^2^ test or Fisher’s test when any expected frequency was lower than five individuals. Linkage disequilibrium (LD) between *HLA-E* alleles and A*01 and A*02 was measured by coefficient D’ (D/Dmax). The confounding effect of gender and age was analyzed by a stratified analysis of the association of *HLA-E* genotypes with the risk of cHL (male, female, <45 years and ≥ 45 years subgroups). Multiple logistic regression was performed to measure the independent contribution of *HLA-E* genetics and other associated *HLA* variables (A*01 and A*02) on the risk of EBV-positive cHL. Data were analyzed using the Statistical Package for the Social Sciences software (SPSS 15, Chicago, IL, USA).

## Results

### Characteristics of cHL patients

One hundred and seventy-five Spanish patients with cHL from Hospital Puerta de Hierro (Madrid) and 400 unmatched healthy individuals from the same area were included in the study. The characteristics of the cHL cohort (Table 1 and S1 Table) were consistent with its epidemiology in Western Europe [36, 37]. The histological distribution was as follows: 60.6% (n = 106) of the cases were NS subtype, followed by a 30.3% (n = 53) with MC histology, 4.6% (n = 8) corresponded to other less frequent subtypes (LD and LR), and 4.6% (n = 8) were not histologically classified.

Consistently with other series [38, 39], 41.7% of the cHL patients were EBV-positive with a bimodal age-incidence curve (data not shown) and the presence of EBV was more frequent in males (79.5% males vs 20.5% females, p = 2.6x10^-5^). Additionally, 64.7% and 30.2% of the MC and NS cases were EBV-positive. The EBV-negative lymphomas presented a unimodal age distribution, were predominantly found in young adults aged <45 years, lacked an association with gender, and the majority had a NS histology (75.5%).

As a replicative series of cHL, we studied 82 patients from a different medical Center in Madrid (MD Anderson Cancer Center, Spain). Clinical and biological data were very similar to the results obtained in our exploratory series, including epidemiology, EBV association and histology, as shown in Table 1 and S1 Table.

### *HLA-E* distribution in controls and cHL patients

Analysis of the *HLA-E* genotype distribution in healthy controls (S2 Table) showed that the majority of the individuals (49.6%) were heterozygous at the dimorphism (E*01:*01*, *01*:*03)*. The major (E*01:*01*) and minor (E*01:*03*) homozygous genotypes were present in 33.3% and 17% of the individuals, respectively. *HLA-E* allelic and genotypic frequencies fell into the range reported for other European ancestry reference populations from *The 1000 genomes project* (http://www.1000genomes.org: CEU, TSI, GBR and FIN) and from other reported population studies [40].

The distribution of the *HLA-E* genotypes in the cHL cohort (regardless of the EBV status), as well as in all the EBV-categorized subgroups, was closely replicated in both series (S2 Table): Hospital Puerta de Hierro (*HPH*) and MD Anderson Cancer Centre (*MDA*). The consistency of the results obtained in the two cohorts supports the validity of our data and, additionally, it permitted to compile and analyze both series together to strengthen the statistical power of the study. The genotype frequencies of *HLA-E* in controls and patients were consistent with Hardy-Weinberg equilibrium.

### HLA-E*01:*01* is a protective factor in cHL and its negative association is mainly derived from EBV-positive cases

In cHL patients (either with a positive or a negative EBV status) E*01:*01* was less frequent compared to controls (individuals with the E*01:*01* allele, 72.4% vs 83%; Table 2; OR 0.5, p = 0.001). To assess the selective effect of *HLA-E* on the risk of EBV-driven lymphomagenesis, EBV-positive and EBV-negative tumors were analyzed separately. Only 67.6% of the EBV-positive cases were homozygous or heterozygous for the E*01:*01* allele, which indicated that E*01:*01*-carriers have a significantly decreased risk of EBV-associated cHL (OR = 0.4, p = 0.0004, Table 2). EBV-negative patients had an intermediate frequency of E*01:*01* (75.8%), and showed a non-significant tendency toward a decreased frequency in comparison to controls (OR = 0.6, p = 0.057). This reduction of E*01:*01* frequency in EBV-negative cases is moderate compared to EBV-positive patients cases and our study lacks sufficient statistical power to determine significant differences between these two subgroups (p = 0.15). These results indicated that the reduction of E*01:*01*-carriers in the global cohort of cHL patients (irrespective of the EBV status) is attributable to differences in the distribution of *HLA-E* genotypes in the EBV-positive subgroup.

**Table 2:** Analysis of the HLA-E*01:*01 allele* as a protective factor in cHL stratified by EBV status.

		E*01:*01* carriers % (N)^a^	OR (95% CI)	χ^2^ p-value
Controls (N = 400)		83.0 (332)		
EBV+ cHL	*HPH* (N = 73)	68.5 (50)	0.5 (0.3–0.8)	0.0038
	*MDA* (N = 35)	65.7 (23)	0.4 (0.2–0.8)	0.011
	*All* (N = 108)	67.6 (73)	0.4 (0.2–0.7)	0.0004
EBV- cHL	*HPH* (N = 102)	75.5 (77)	0.63 (0.4–1.1)	0.081
	*MDA* (N = 47)	76.6 (36)	0.7 (0.3–1.4)	0.28
	*All* (N = 149)	75.8 (113)	0.64 (0.4–1.0)	0.057
Total cHL	*HPH* (N = 175)	72.6 (127)	0.5 (0.4–0.8)	0.0042
	*MDA* (N = 82)	71.6 (59)	0.5 (0.3–0.9)	0.02
	*All* (N = 257)	72.4 (186)	0.5 (0.4–0.8)	0.001

HPH: patients from Hospital Puerta de Hierro, MDA: patients from MD Anderson Cancer Center

OR: odds ratio; CI, confidence interval

^a^ Genotypes with the protective allele (homozygous E*01:*01* and heterozygous)

We analyzed the potential effect of the E*01:*01* allele dose on the decreased risk of EBV-positive cHL. Similar OR values were obtained for carriers of one (OR = 0.4, p = 0.002) or two copies (OR = 0.4, p = 0.002) of E*01:*01* compared to donors lacking the protective allele, indicating that the risk of suffering from EBV-positive cHL does not depend on the E*01:*01* allele dosage.

### The association of HLA-E*01:*01* with EBV-positive cHL is not influenced by age or gender

We stratified the series by gender and age, variables previously associated with EBV-associated cHL, to investigate their possible confounding effect on the contribution of the *HLA-E* genotype to the risk of developing EBV-associated cHL. The subgroup analysis showed that E*01:*01* was similarly associated in males and females (OR = 0.4, p = 0.0037 and OR = 0.4, p = 0.028; respectively) and in <45 years old (OR = 0.5, p = 0.021) versus ≥45 years old (OR = 0.4, p = 0.009) categories (Table 3). This analysis confirmed that the negative association between the E*01:*01* allele and the risk of EBV-positive cHL was not influenced by age or gender. Similarly, stratifications of the EBV-positive cHL series by histology and other clinical parameters (disease stage, presence of bulky tumor and survival) did not reveal any differences in the distribution of the *HLA-E* genotypes (data not shown).

**Table 3:** Stratification of HLA-E*01:*01* frequency for age and gender.

Variable	Subjects	E*01:*01* allele % (N)	OR (95% CI)	χ^2^ p-value
*Gender*				
Male	EBV+ cHL (N = 78)	73.1 (57)	0.4 (0.2–0.74)	0.0037
	Controls (N = 193)	87.6 (169)		
Female	EBV+ cHL (N = 30)	53.3 (23)	0.4 (0.21–0.92)	0.028
	Controls (N = 212)	78.7 (167)		
*Age (years)*				
<45	EBV+ cHL (N = 56)	69.6 (39)	0.5 (0.24–0.9)	0.021
	Controls (N = 243)	83.1 (202)		
≥45	EBV+ cHL (N = 55)	65.4 (34)	0.4 (0.2–0.8)	0.009
	Controls (N = 160)	82.6 (132)		

OR: odds ratio; CI, confidence interval

### Analysis of *HLA-E/HLA-A* interaction: Identification of *HLA-E/HLA-A* phenotypes associated to the EBV-positive cHL susceptibility

*HLA* alleles A*01 and A*02 have been reported to associate with an increased and a reduced risk of EBV-positive cHL, respectively [41, 42]. In our study, A*01 and A*02 in healthy controls had similar frequencies to those reported for Spanish population (A*01: 16.8% and A*02: 51.9%) [43] and consistent with previous works, in the EBV-positive cHL cohort, A*01 is of risk (43.2%, OR = 3.8, p = 2.3x10^-8^) whilst A*02 is protective (31.9%, OR = 0.4, p = 0.001).

Given the strong LD in the *HLA* region and the vicinity of *HLA-A* and -*E* loci, we analyzed the genetic association of *HLA-E* alleles and A*01 and A*02, in order to study their interaction and relative contribution to EBV-positive cHL. The LD analysis within our control samples showed that the predisposing A*01 allele was in strong positive LD with the protective E*01:*01* allele (D’ = 0.65, p = 0.0082) and in weaker, non-significant negative LD with the risk E*01:*03* allele (D’ = −0.13, p = 0.096). On the contrary, the protective A*02 presented a random association with the protective E*01:*01* (D’ = −0.05, p = 0.64) and a weak LD with the risk E*01:*03* (D’ = 0.15, p = 0.026).

Strikingly, in patients, *HLA-A* and *HLA-E* alleles with opposed effects on the risk of the EBV-related cHL had a lower LD compared to those described for controls. We found a decreased non-significant LD between the protective E*01:*01* and the predisposing A*01 (D’ = 0.34, p = 0.064) and no association between the risk E*01:*03* and the protective A*02 (D’ = 0.02, p = 0.86). These reduced LD between *HLA-A* and *HLA-E* alleles with a confronting influence on EBV-associated cHL risk would indicate a selection of *HLA* phenotypes composed by predisposing *HLA-A* and *HLA-E* alleles in EBV-positive cHL patients.

A subgroup analysis stratified by *HLA-A* alleles revealed a similar distribution of E*01:*01* in A*01-carriers (OR = 0.3, p = 0.029) and in A*01-negative donors (OR = 0.4, p = 0.003); and, similarly, in A*02-positive (OR = 0.4, p = 0.036) or A*02-negative subjects (OR = 0.6, p = 0.088). In A*01 A*02 double negative subjects, E*01:*01* also presents comparable frequencies, but sample size is not large enough to detect significant differences (80.5% vs. 68.6%; OR = 0.5, p = 0.17). These results indicate that A*01 and A*02 alleles are not confounding factors influencing the protective association between the E*01:*01* allele and the risk of EBV-positive cHL.

A multiple logistic regression analysis was performed to further assess the possible influence of A*01 and A*02 on the association of *HLA-E* to the risk of EBV-related cHL (Table 4). After adjusting by presence of HLA-A*01 and A*02, E*01:*01* remained as a significant protective factor. This analysis confirmed that E*01:*01* is negatively associated with the risk of EBV-positive cHL (OR = 0.4, p = 0.001) and this association is independent of other known *HLA* factors that impact on the risk of EBV-related cHL.

**Table 4:** Multivariate logistic regression analysis: E*01:*01* contributes independently of other *HLA* factors to the risk of EBV+ cHL.

Variable	OR	95% CI	p-value
E*01:*01 positive*	**0.4**	**0.2–0.7**	**0.001**
A*01 positive	3.9	2.3–6.5	6.7x10^-7^
A*02 positive	0.4	0.2–0.6	0.001

OR: odds ratio; CI, confidence interval

Finally, we examined the cumulative effect of the *HLA-A* and-*E* polymorphism on the risk of EBV-positive cHL through the comparison of protective phenotypes (E*01:*01* ^*+ve*^, A*01 ^*-ve*^ or/and A*02 ^*+ve*^) to all other phenotypes or to its opposite risk phenotype (E*01:*01* ^*-ve*^, A*01 ^*+ve*^ or/and A*02 ^*-ve*^) (Table 5). Protective *HLA-E-HLA-A* combinations were dominant in healthy controls but clearly underrepresented in EBV-associated cHL (E*01:*01* ^*+ve*^ A*01 ^*-ve*^: 67.2% vs 35.8%, E*01:*01* ^*+ve*^ A*02 ^*+ve*^: 42.6% vs 20.9% and E*01:*01* ^*+ve*^ A*01 ^*-ve*^ A*02 ^*+ve*^: 36.1% vs 9.5%). On the contrary, the reciprocal risk phenotypes had very low rates in healthy controls and were increased in EBV-associated cHL (E*01:*01* ^*-ve*^ A*01 ^*+ve*^: 1% vs 8.4%, E*01:*01* ^*-ve*^ A*02 ^*-ve*^: 7.8% vs 16.8% and E*01:*01* ^*-ve*^ A*01 ^*+ve*^ A*02 ^*-ve*^: 0.3% vs 5.3%). In summary, comparison of the frequencies of the protective *HLA-A-HLA-E* phenotypes to the frequencies of any other genotypes or their opposite risk phenotypes showed a marked decrease on the risk of EBV-associated cHL, demonstrating an additive effect of *HLA-E* and *HLA-A* genetics on protection against EBV-positive cHL (e.g. OR = 0.013 for the E*01:*01* ^*+ve*^ A*01 ^*-ve*^ A*02 ^*+ve*^ phenotype in comparison with its reciprocal, p = 2.9x10^-5^).

**Table 5:** Analysis of the combined effect of HLA-E*01:*01*, A*01 and A*02 on the risk of EBV-associated cHL.

Protective phenotype	EBV+ cHL %(N)	Controls %(N)	OR (95% CI)	p-value
E*01:*01* ^*+ve*^ A*01 ^*-ve*^	35.8 (34)	67.2 (268)		
vs opposite phenotype	8.4 (8)	1.0 (4)	0.06 (0.02–0.22)	0.0004
vs all other phenotypes	64.2 (61)	32.8 (131)	0.27 (0.17–0.44)	<10^−8^
E*01:*01* ^*+ve*^ A*02 ^*+ve*^	20.9 (19)	42.6 (170)		
vs opposite phenotype	16.8 (16)	7.8 (31)	0.22 (0.10–0.47)	3.4x10^-5^
vs all other phenotypes	79.1 (72)	57.4 (229)	0.34 (0.20–0.58)	4.3x10^-5^
E*01:*01* ^*+ve*^ A*01 ^*-ve*^ A*02 ^*+ve*^	9.5 (9)	36.1 (144)		
vs opposite phenotype	5.3 (5)	0.3 (1)	0.013 (0.001–0.12)	2.9x10^-5^
vs all other phenotypes	90.5 (80)	63.9 (255)	0.18 (0.09–0.36)	<10^−8^

OR: odds ratio; CI, confidence interval

^+ve^: positive

^-ve^: negative

## Discussion

Inherited variations in host immune responses have long been associated with susceptibility or protection against disease, including tumors and infections. We analyzed the contribution of *HLA-E* genetics to the susceptibility of developing EBV-driven cHL in a case-control study. Our results showed a reduced frequency of E*01:*01* in EBV-positive cHL cases compared to controls. This difference indicated a protective effect of this variant─ E*01:*01*-positive individuals would have more than a 50% reduction in the risk of developing EBV-associated cHL. The validity of this genetic association is strengthened by the replication of the *HLA-E* distribution in a supplementary series of cHL patients in the same geographic region. We also found that the association of *HLA-E* with EBV-positive cHL is not linked to other reported demographic factors associated with this disease such as age and gender. The relationship of cHL resistance to the E*01:*01* allele did not show a dose effect, likely implying a dominant influence of this variant on protection against the disease.

A potential biological rationale for the genetic association with EBV-associated lymphomas described herein could be the influence of *HLA-E* polymorphism on CD94/NKG2A signaling in NK cells. In this model, it may be speculated that lower expression of HLA-E*01:01 in EBV-infected cells, as compared with E*01:03, could confer an attenuated NKG2A-mediated inhibition of NK or CD8+ T cell killing. This phenomenon could be translated into an enhanced EBV-directed cytotoxic response in E*01:01 carriers that could contribute to protection against a malignant course of EBV infection. Another hypothesis is that E*01:01 could more efficiently present EBV-derived peptides to CD8+ T lymphocytes than E*01:03, generating an increased cytotoxic T cell response to EBV infection.

In EBV-negative cHL, the frequency of E*01:*01* was intermediate between healthy controls and EBV+ cases. Although we did not find significant differences because the reduction of the frequency of the E*01:*01* allele was not as pronounced as that in the EBV+ group, the results could also have a potential biological significance. HLA-E is expressed at high levels by some tumors [44–46], including neoplastic and microenvironment cells in some cHL patients [47]. The expression and function of HLA-E in cancer cells is not fully understood, but it may represent a mechanism of tolerance that could be influenced by *HLA-E* polymorphism. Further studies with larger cohorts should be performed to better estimate the relevance of *HLA-E* genetics in EBV-negative cHL.

One limitation of our study is that the control series could not be monitored for antibodies against EBV. To assess how EBV-serostatus would impact on our results, we estimated, from age-adjusted EBV-seroprevalence data of our Institution (Dr. Portero, F, Microbiology Department; personal communication), a 4% of EBV IgG seronegative controls in our cohort. With this seronegativity rate, and even in the worst scenario for our conclusions (i.e. subtracting 4% [N = 25] supposed seronegatives only from the E*01:01 carrier group), we estimated an irrelevant influence of EBV-seronegative subjects on our results (data not shown)

The influence of the *HLA-E* locus on the genetic background of EBV-induced tumors has also been studied in nasopharyngeal carcinoma (NPC), an EBV-associated malignancy with a restricted racial and geographical distribution. In Thai NPC patients, a homozygous E*01:*03* genotype was associated with an increased risk of disease, i.e., E*01:*01*-carriers were, as in this study, protected for EBV-tumorigenesis [48]. However, although a similar trend was found in Tunisian patients with NPC, neither *HLA-E* allele was found to have major effects on NPC susceptibility or progression [49].

Previous investigations on the association of the *HLA class I* region with EBV-associated cHL pointed to *HLA-A1* and *A2* being positively and negatively associated, respectively, with EBV+ cHL [47, 48]. The *HLA-E* locus is ~540 kb centromeric to *HLA-A* (http://vega.sanger.ac.uk). The physical proximity of the two genes raised the question of whether the relationship between *HLA-E* and EBV-positive cHL could be secondary to an association with *HLA-A* or vice versa. Interestingly, we found that the association of *HLA-E* with EBV-positive cHL is independent of those of A*01 or A*02, which were replicated in our study. Despite the extensive amount of studies assessing the LD in MHC region, few of them include data on *HLA-E* polymorphism. These studies describe an essentially random association of their alleles, probably due to the presence of recombination hot spots [24] but also, some conserved *HLA-A*-*HLA-E* haplotypes, including the combination of the EBV-positive Chl protective E*01:*01* and the predisposing A*01 [50]. Here, we found also this association between A*01 and E*01:*01* in healthy individuals. This haplotype, contradictory in terms of influence on EBV-positive cHL risk, presents a lower LD among EBV-positive cHL patients in whom it is underrepresented. An analysis integrating the effect of E*01:*01*, A*01 and A*02 revealed the existence of protective phenotypes by which E*01:*01*-positive, A*01-negative and A*02-positive individuals would have more than 70 times less risk of developing EBV-associated cHL than the opposite high-risk phenotype (E*01:*01*-negative, A*01-positive and A*02-negative). The high frequencies of the protective *HLA-A-HLA-E* phenotypes and the low rates of the opposite risk phenotypes in healthy individuals could indicate an EBV selective pressure shaping *HLA-A* and *HLA-E* polymorphism. Our results suggest a potential use of combined *HLA-A* and-*E* typing as a biomarker of increased risk of EBV-positive cHL

EBV-positive cHL presents an intrincate epidemiology, and the overall incidence rates vary greatly in different regions of the world [36]. The origin of this geographic discrepancy is unknown, but even when modifiable factors such as lifestyle or the environment are controlled for epidemiological studies, some variability remains across the ethnic groups [51]. These persistent differences in susceptibility to EBV-associated cHL among human populations might be partially explained by polymorphic genetic systems that show ethnic variation in the rates of allelic prevalence, such as *HLA-E* and *HLA-A*.

The association of the *HLA-E* genetic diversity with the susceptibility of EBV-driven cHL suggests a role for the HLA-E molecule in the etiopathogenesis of the disease. These findings may contribute to the understanding of the influence of host genetics on EBV-related lymphomas.

## Supporting Information

S1 Table Clinical and histological features of classic Hodgkin lymphoma subjects. (DOCX)

S2 Table Distribution of *HLA-E* genotypes in controls and patients. (DOCX)
