# Supplementary material for: HLA Allele E*01:01 Is Associated with a Reduced Risk of EBV-Related Classical Hodgkin Lymphoma Independently of HLA-A*01/*02
Source: PLoS One. 2015 Aug 11;10(8):e0135512. doi: 10.1371/journal.pone.0135512 (PMC4532421; doi:10.1371/journal.pone.0135512)
Supplement: S1 Table — (DOCX) [file pone.0135512.s001.docx]

**Table S1**: Clinical and histological features of classic Hodgkin lymphoma subjects

| % (N) | |  | All cHL | |  | EBV+ cHL | |  | EBV- cHL | |
| --- | --- | --- | --- | --- | --- | --- | --- | --- | --- | --- |
|  |  |  | *HPH* | *MDA* |  | *HPH* | *MDA* |  | *HPH* | *MDA* |
|  |  |  |  |  |  |  |  |  |  |  |
| *Total* | |  | 100 (175) | 100 (82) |  | 40.8 (73) | 42.7 (35) |  | 58.3 (102) | 53.7 (47) |
| *Gender* | |  |  |  |  |  |  |  |  |  |
| Male | |  | 61.1 (107) | 57.3 (47) |  | 79.5 (58) | 57.1 (20) |  | 48.0 (49) | 57.4 (27) |
| Female | |  | 38.9 (68) | 42.7 (35) |  | 20.5 (15) | 42.9 (15) |  | 52.0 (53) | 42.6 (20) |
| *Age (years)* | |  |  |  |  |  |  |  |  |  |
| <45 years | |  | 69.7 (122) | 64.6 (53) |  | 52.1 (38) | 51.4 (18) |  | 82.4 (84) | 74.5 (35) |
| >45 years | |  | 30.3 (53) | 35.4 (29) |  | 47.9 (35) | 48.6 (17) |  | 17.6 (18) | 25.5 (12) |
| *Histological subtype* | |  |  |  |  |  |  |  |  |  |
| NS | |  | 60.6 (106) | 61.0 (50) |  | 43.8 (32) | 45.7 (10) |  | 72.5 (74) | 72.3 (34) |
| MC | |  | 30.3 (53) | 29.3 (24) |  | 45.2 (33) | 37.1 (13) |  | 19.6 (20) | 23.4 (11) |
| LD | |  | 2.3 (4) | 0 |  | 2.7 (2) | 0 |  | 2.0 (2) | 0 |
| LR | |  | 2.3 (4) | 3.7 (3) |  | 2.7 (2) | 8.6 (3) |  | 2.0 (2) | 0 |
| NC | |  | 4.6 (8) | 6.1 (5) |  | 5.5 (4) | 8.6 (3) |  | 3.9 (4) | 4.3 (2) |
| *Disease stage* | |  |  |  |  |  |  |  |  |  |
| Early | |  | 41.7 (73) | 14.6 (12) |  | 37.0 (27) | 14.3 (5) |  | 45.1 (46) | 14.9 (7) |
| Advanced | |  | 55.4 (97) | 81.3 (67) |  | 61.6 (45) | 80 (28) |  | 51.0 (52) | 83.0 (38) |
| ND | |  | 2.9 (5) | 3.7 (3) |  | 1.4 (1) | 5.7 (2) |  | 3.9 (4) | 2.1 (1) |
| *Bulky* |  |  |  |  |  |  |  |  |  |  |
| Yes | |  | 21.8 (39) | 30.5 (25) |  | 16.4 (12) | 22.9 (8) |  | 25.5 (27) | 36.1 (17) |
| No | |  | 67.0 (120) | 64.6 (53) |  | 67.1 (49) | 71.4 (25) |  | 67.0 (71) | 59.6 (28) |
| ND | |  | 11.2 (20) | 4.9 (4) |  | 16.4 (12) | 5.7 (2) |  | 7.5 (8) | 4.3 (2) |
| *Status* |  |  |  |  |  |  |  |  |  |  |
| Dead | |  | 22.3 (40) | 7.3 (6) |  | 34.2 (25) | 17.1 (6) |  | 14.2 (15) | 0 |
| Alive | |  | 73.7 (132) | 85.4 (70) |  | 60.3 (44) | 74.3 (26) |  | 83.0 (88) | 93.6 (44) |
| ND | |  | 3.9 (7) | 7.3 (6) |  | 5.5 (4) | 8.6 (3) |  | 2.8 (3) | 6.4 (3) |
|  |  |  |  |  |  |  |  |  |  |  |

HPH: patients from the Hospital Puerta de Hierro, MDA: patients from MD Anderson Cancer Center

NS: nodular sclerosis, MC: mixed cellularity, LD: lymphocyte-depletion, LR: lymphocyte-rich, NC: not classified. ND: not determined
