# Supplementary material for: HLA Allele E*01:01 Is Associated with a Reduced Risk of EBV-Related Classical Hodgkin Lymphoma Independently of HLA-A*01/*02
Source: PLoS One. 2015 Aug 11;10(8):e0135512. doi: 10.1371/journal.pone.0135512 (PMC4532421; doi:10.1371/journal.pone.0135512)
Supplement: S2 Table — (DOCX) [file pone.0135512.s002.docx]

**Table S2**. Distribution of *HLA-E* genotypes in controls and patients

|  | | *HLA-E* genotypes %(N) | | | | | | | | |
| --- | --- | --- | --- | --- | --- | --- | --- | --- | --- | --- |
|  |  |  |  |  |  |  |  |  | |  |
|  |  | *Homozygous E*01:01* |  | *Heterozygous E*01:01, 01:03* |  | *Homozygous*  *E*01:03* | | |  |  |
|  |  |  |  |  |  |  | | |  |  |
| Controls (N=400) | | 33.0 (132) |  | 50.0 (200) |  | 17.0 (68) | | |  |  |
|  |  |  |  |  |  |  | | |  |  |
|  |  |  |  |  |  |  | | |  |  |
| EBV+ cHL | *HPH* (N=73) | 23.3 (17) |  | 45.2 (33) |  | 31.5 (23) | | |  |  |
|  | *MDA* (N=35) | 31.4 (11) |  | 34.3 (12) |  | 34.3 (12) | | |  |  |
|  | All (N=108) | 25.9 (28) |  | 41.7 (45) |  | 32.4 (35) | | |  |  |
|  |  |  |  |  |  |  | | |  |  |
| EBV- cHL | *HPH* (N=102) | 21.6 (22) |  | 53.9 (55) |  | 24.5 (25) | | |  |  |
|  | MDA (N=47) | 21.3 (10) |  | 55.3 (26) |  | 23.4 (11) | | |  |  |
|  | All (N=149) | 21.5 (32) |  | 54.4 (81) |  | 24.2 (36) | | |  |  |
|  |  |  |  |  |  |  | | |  |  |
| Total cHL | *HPH* (N=175) | 22.3 (39) |  | 50.3 (88) |  | 27.4 (48) | | |  |  |
|  | *MDA* (N=82) | 25.6 (21) |  | 46.3 (38) |  | 28.0 (23) | | |  |  |
|  | All (N=257) | 23.3 (60) |  | 49.0 (126) |  | 27.6 (71) | | |  |  |
|  |  |  |  |  |  |  |  |  | |  |

HPH: patients from the Hospital Puerta de Hierro, MDA: patients from MD Anderson Cancer Center
